# Caspase-8-dependent autophagy regulates neutrophil infiltration in oral squamous cell carcinoma

**DOI:** 10.1073/pnas.2406944121

**Published:** 2024-12-03

**Authors:** Miguel Bernabé-Rubio, Fiona M. Watt

**Affiliations:** ^a^Centre for Gene Therapy and Regenerative Medicine, School of Basic and Medical Biosciences, King’s College London, London SE1 9RT, United Kingdom; ^b^Directors’ Unit, European Molecular Biology Laboratory Heidelberg, Heidelberg 69117, Germany

**Keywords:** epithelia, cancer, neutrophils, autophagy

## Abstract

Oral squamous cell carcinoma (OSCC) is a subtype of head and neck cancer that arises in the multilayered epithelia of the mouth and lips. Although inactivating mutations in *CASP8* are frequently found in human OSCC their role in the disease is unknown. To investigate this, we deleted *Casp8* in the oral epithelium of adult mice. Loss of Caspase-8 resulted in defects in the tongue epithelial barrier and triggered a neutrophil-rich immune infiltrate distinct from that observed on epidermal *Casp8* deletion. Oral *Casp8* deletion led to activation of autophagy. Inhibition of autophagy partially rescued epithelial integrity in *Casp8*^−/−^ mice, while induction of autophagy in wild type mice resulted in oral barrier defects and excessive neutrophil infiltration. On treatment with the carcinogen 4-nitroquinoline-1-oxide *Casp8*^−/−^ mice showed increased susceptibility to developing oral tumors. Depletion of neutrophils reduced tumor incidence, which correlated with a reduction in reactive oxygen species and decreased epithelial DNA damage. Our findings establish a functional link between epithelial integrity, autophagy, and the tumor immune microenvironment, placing Caspase-8 at the center of these processes.

## Significance

Oral squamous cell carcinoma is genetically heterogeneous and has a poor 5-y survival rate. To explore the role of inactivating mutations in Caspase-8, we deleted the gene in the oral cavity of mice. This resulted in an epithelial barrier defect and neutrophil infiltrate, both of which were linked to the induction of autophagy in epithelial cells. On treatment with a chemical carcinogen deletion of Caspase-8 resulted in increased reactive oxygen species and tumor susceptibility. Ablation of neutrophils reduced tumor formation. Our results shed light on the functional significance of Caspase-8 in oral cancer.

Head and neck squamous cell carcinoma comprises a diverse group of epithelial tumors originating from the upper aerodigestive tract (1). Oral squamous cell carcinoma (OSCC), a subtype of the disease, is one of the most common cancers worldwide (2). Despite advances in treatment and diagnosis, the 5-y survival rate is only 50% (1). OSCC mainly occurs in the tongue but can also involve the floor of the mouth, gingiva, lip, cheek, and palate. OSCCs are strikingly heterogeneous at the genetic and cellular levels (3) and exhibit diverse stromal and immune microenvironments. *CASP8* is frequently mutated in OSCC (3–5), and *CASP8* mutations are linked to poor survival. However, the role of these mutations in cancer is incompletely characterized.

Caspase-8 is an aspartate-specific cysteine protease that initiates apoptotic cell death (6). This process, referred to as extrinsic apoptosis, involves the binding of death receptors with their cognate ligands. Given the key role of Caspase-8 in apoptosis, *Casp8* has typically been considered a tumor suppressor gene (7). Besides its well-established role in apoptotic cell death, Caspase-8 is involved in epithelial differentiation, integrin signaling, and inflammation (8–10). In recent years, macroautophagy, hereafter referred to as autophagy, has emerged as a regulator of inflammation. Inhibition of Caspase-8 induces autophagy-dependent apoptosis (11) and T cells lacking Caspase-8 activity show hyperactive autophagic signaling (12).

The oral cavity is continuously exposed to antigens (13). However, the oral mucosal barrier is understudied compared to the epithelial barrier of other organs such as the gastrointestinal tract or skin, and the functional role of different immune cell subpopulations in oral homeostasis and cancer is poorly understood. Under steady state conditions, neutrophils are the immune cell type with highest representation in human oral mucosa (14), which contrasts with healthy skin, in which neutrophils are rarely found (15). Neutrophils continuously extravasate into the oral cavity where they play a role in microbial surveillance. Whether the extravasated neutrophils have additional functional roles in the oral cavity and whether these are influenced by *Casp8* inactivation remains unclear. Recently, there have been multiple reports that neutrophils can promote tumor progression in a range of cancers (16, 17). Neutrophils can induce oxidative DNA damage in epithelial cells, thereby contributing to oncogenic mutations.

In this study, we sought to determine the impact of loss of *Casp8* on oral epithelial homeostasis and carcinogen-induced OSCC. We report that downregulation of *Casp8* affects epithelial barrier formation leading to an immune cell infiltrate whose cell content is tissue specific. Furthermore, neutrophils infiltrating the tongue epithelium act as tumor promoters.

## Results

### Caspase-8 Regulates Skin and Tongue Epithelial Barriers.

The basal layer of multilayered epithelia expresses Keratin 14 (Krt14) and Keratin 5 (Krt5), and therefore basal keratinocytes of transgenic mice can be targeted using the Krt14 or Krt5 promoters. The resulting skin phenotype of *Casp8* deletion in basal keratinocytes has been characterized previously (18–20). Epidermal knockouts obtained by crossing mice carrying the floxed Caspase-8 allele (Casp8 fl/fl) with mice expressing Krt14Cre have early onset severe epidermal hyperproliferation and inflammation, and fail to thrive (19). For this reason, we selectively deleted *Casp8* via Tamoxifen-inducible Cre (Krt14CreER) through injection of Tamoxifen in adult mice.

**Fig. 1.**
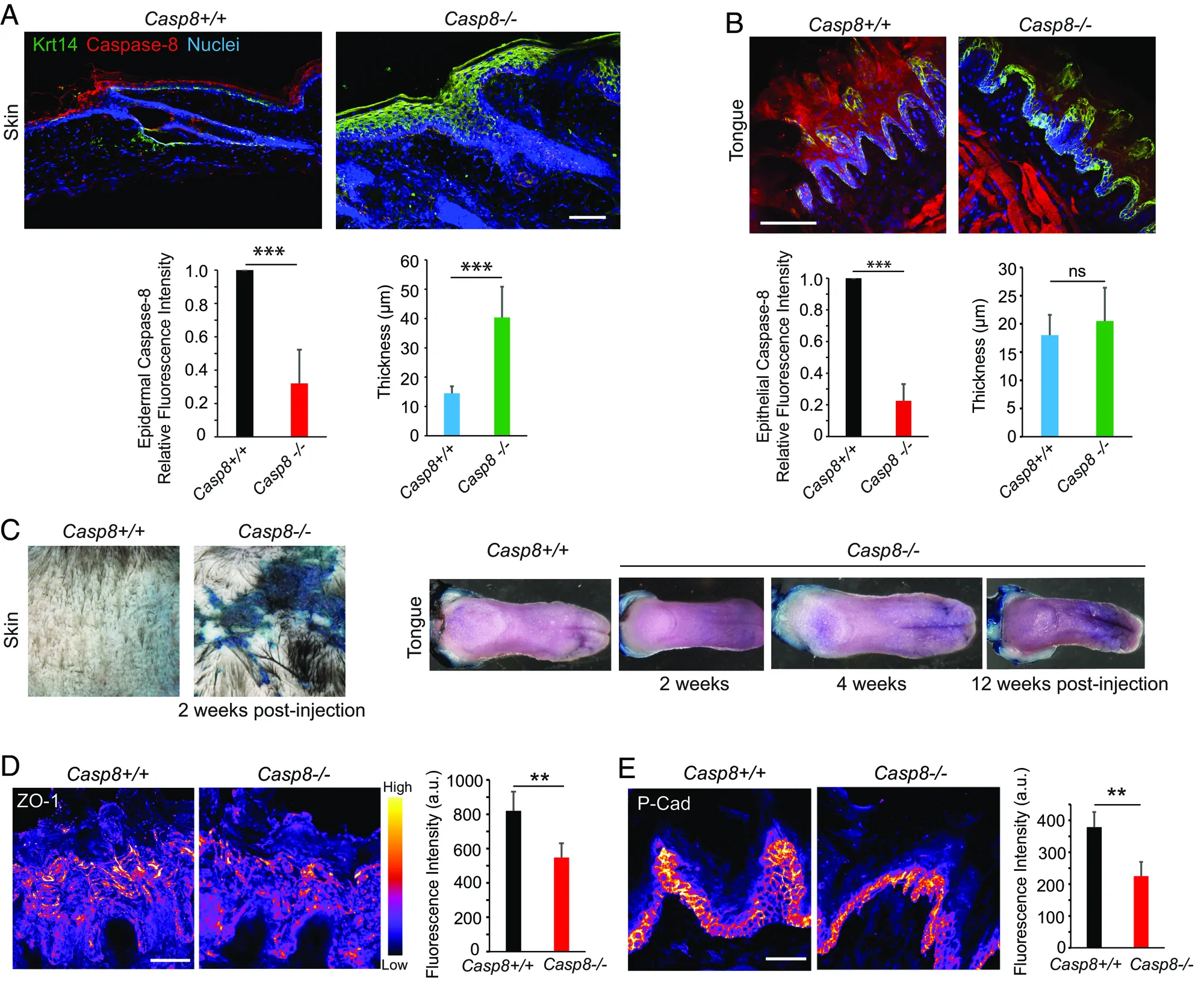
Skin and tongue barriers are regulated by Caspase-8. (*A* and *B*) Representative skin (*A*) and tongue (*B*) sections stained for Krt14 and Caspase-8 (*Upper*). Nuclei are visualized with DAPI staining. Note Caspase-8 labeling of the connective tissue in *Casp8^−/−^* tongue sections. Bar graphs showing Caspase-8 relative epithelial protein levels per 0.01 mm^2^ epithelium and epithelial thickness (*Lower*). Data are the mean ± SD *n* = 3 mice per condition. To quantify Caspase-8 levels the background epithelial fluorescence of sections labeled with second antibody alone was first subtracted; values above zero on *Casp8* deletion reflect background variation between the different samples. Epithelial thickness was determined in Krt14 labeled sections. (Scale bars, 40 µm.) (*C*) Skin (*Left*) and tongue (*Right*) stained with toluidine blue. The skin was analyzed at 2 wk after tamoxifen injection, whereas the tongue was analyzed at 2, 4, and 12 wk. (*D* and *E*) Representative tongue sections were stained with ZO-1 (*D*) and P-Cad antibodies (*E*) and color-coded for signal intensity with ImageJ (*Left*). Note the disruption of the cell junctions upon *Casp8* deletion. Bar graphs showing total fluorescence intensity per 0.01 mm^2^ epithelium of ZO-1 (*D*) and P-Cad (*E*) of *Casp8*^+/+^ and *Casp8*^−/−^ mice (*Right*). Data are the mean ± SD *n* = 3 mice per condition. (Scale bars, 20 µm.) Two-tailed Student’s unpaired *t* test was used to determine statistical significance in *A*, *B*, *D*, and *E*. *P < 0.05; **P < 0.01; ***P < 0.001; ns, not significant.

Specific deletion of *Casp8* in keratinocytes of the epidermis and oral cavity was confirmed by antibody staining and qPCR (Fig. 1 *A* and *B* and *SI Appendix*, Fig. S1*A*). In control mice, Caspase-8 distributed mainly in the suprabasal layers of the epidermis (Fig. 1*A*), consistent with previous reports (18). The tongue epithelium also showed a suprabasal distribution of Caspase-8 (Fig. 1*B*).

As expected, on *Casp8* deletion there was an increase in epidermal thickening (Fig. 1*A*) and increased proliferation in the epidermis as judged by Ki67 staining (*SI Appendix*, Fig. S1 *B* and *C*). However, proliferation remained unaltered in the tongue epithelium in the absence of Casp8 (*SI Appendix*, Fig. S1 *B* and *C*). In contrast to the epidermis, the Krt14+ basal compartment did not expand in *Casp8*^−/−^ tongue epithelium (Fig. 1*B*) and we observed no histological abnormalities (*SI Appendix*, Fig. S1*D*). Additionally, staining with antibodies to Filaggrin suggested that the differentiation program was unaffected, although we could not rule out subtle abnormalities (*SI Appendix*, Fig. S1*E*).

Although loss of Caspase-8 did not affect the overall tongue architecture it did compromise the epithelial barrier. Caspase-8 activity is required for the maintenance of epidermal barrier function (21). Using penetration of the dye toluidine blue we confirmed the defect in the epidermal barrier (Fig. 1*C*). We also observed a sustained barrier defect in the tongue of *Casp8*^−/−^ mice (Fig. 1*C*). In skin, defects in epithelial integrity were evident after 2 wk of *Casp8* deletion, whereas in the tongue these effects were not evident until 4 wk post-Tamoxifen injection. This difference could be due to the different degrees of stratification in skin and tongue epithelia, the latter being substantially thicker.

The defect in the tongue barrier correlated with disruption in tight junctions and adherens junctions, as indicated by ZO-1, and P-Cad and E-Cad staining, respectively. Total protein levels were reduced (*Right*-hand panels) and cellular localization (*Left*-hand panels) was disrupted (Fig. 1 *D* and *E* and *SI Appendix*, Fig. S1*F*). These results are in line with reports that Caspase-8 catalytic activity is important in maintaining the intestinal barrier (21), that human OSCC lines with inactivating mutations in *CASP8* have reduced intercellular adhesion (3) and that depletion of *Casp8* results in similar pathologies to those observed in tissues where catalytic activity is impaired (22).

### Differences in the Immune Infiltrate of *Casp8*^−/−^ Skin and Tongue Epithelia.

**Fig. 2.**
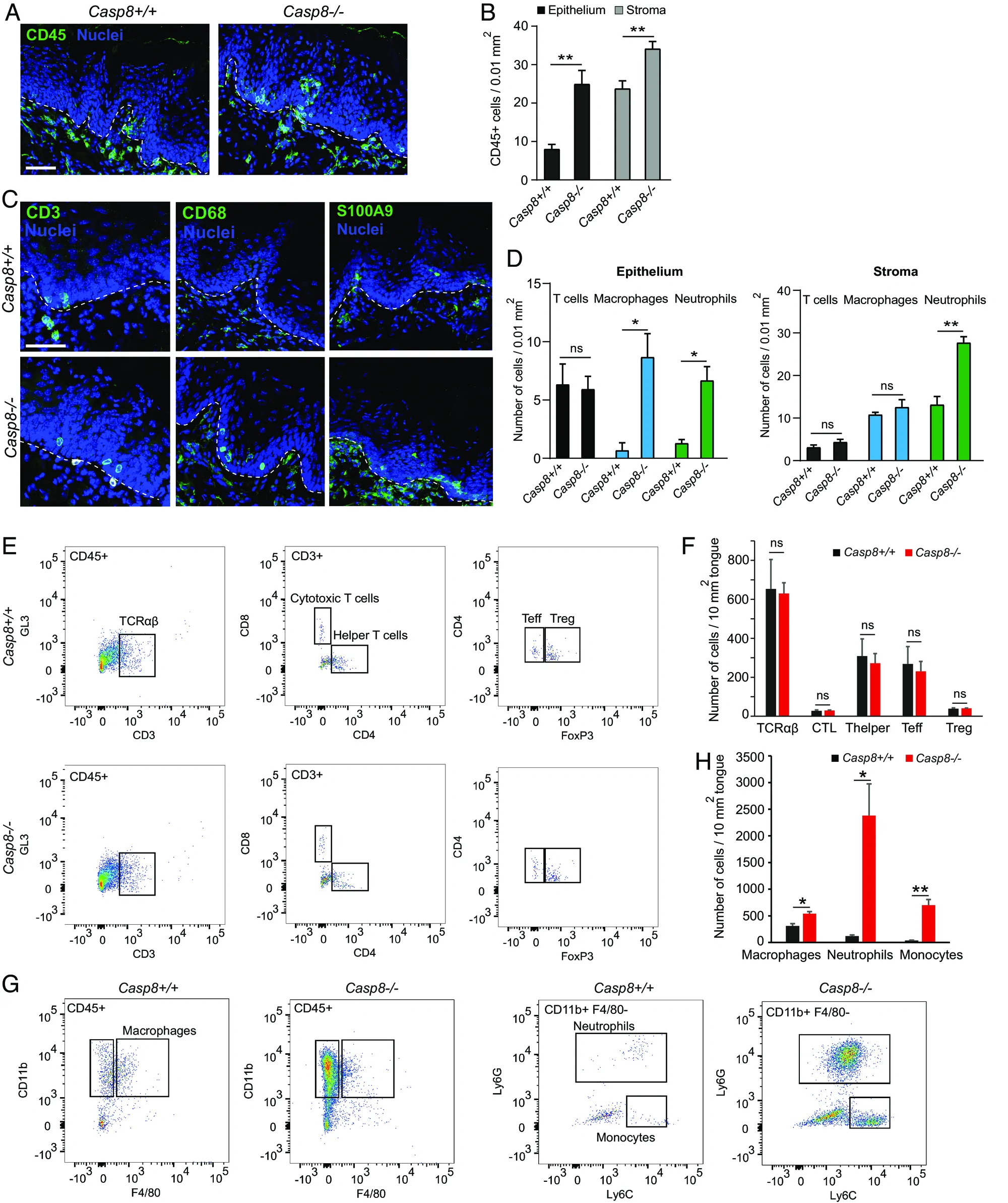
Immune infiltrate content is tissue-dependent upon downregulation of *Casp8*. (*A*) Representative tongue sections stained for CD45. (Scale bar, 20 µm.) (*B*) Bar graphs showing the number of CD45+ cells per 0.01 mm^2^ in the epithelium and the stroma. Data are the mean ± SD n = 5 mice per condition. (*C*) Representative tongue sections stained for CD3 (T-cells), CD68 (macrophages), and S100A9 (neutrophils). (Scale bar, 40 µm.) (*D*) Bar graphs showing the number of CD3+, CD68+, and S100A9+ cells in the epithelium (*Left*) and stroma (*Right*). Data are the mean ± SD n = 5 mice per condition. (*E*) Representative flow cytometric analysis showing αβT-cells (CD3^+^ GL3^-^), cytotoxic T cells (CD3^+^ CD8^+^), helper T cells (CD3^+^ CD4^+^), effector T cells (CD3^+^ CD4^+^ FoxP3^−^), and Tregs (CD3^+^ CD4^+^ FoxP3^+^) in the tongue of *Casp8*^−/−^ and control mice. Note that γδT-cells (CD3^+^ GL3^+^) were not detected. (*F*) Bar graphs showing the number of cells per 10 mm^2^ analyzed in *E*. Data are the mean ± SEM. n = 3 mice per condition. (*G*) Representative flow cytometric analysis showing macrophages (CD11b^+^ F4/80^+^), neutrophils (CD11b^+^ Ly6G^+^), and monocytes (CD11b^+^ Ly6C^+^) in the tongue of *Casp8*^−/−^ and control mice. (*H*) Bar graphs showing the number of cells analysed in (*G*). Data are the mean ± SD. n = 3 mice per condition. Data are the mean ± SEM. n = 3 mice per condition. Two-tailed Student’s unpaired *t* test was used to determine statistical significance in *B*, *D*, *F*, and *H*. *P < 0.05; **P < 0.01; ns, not significant. The dashed lines demarcate epithelial-stroma boundaries.

Having observed that there were epithelial barrier defects on *Casp8* deletion, we examined whether immune cells could infiltrate the tongue epithelium, and, if so, whether the infiltrate was similar to that of the epidermis. Staining with the panleukocyte marker CD45 showed an increase in the immune infiltrate in both epithelia of *Casp8*-depleted mice (Fig. 2 *A* and *B* and *SI Appendix*, Fig. S2 *A* and *B*). In the tongue epithelium, the number of CD3+ T cells remained unchanged (Fig. 2 *C* and *D*). There was an increase in the number of macrophages and neutrophils; however, in the stroma, only the number of neutrophils was significantly elevated (Fig. 2 *C* and *D*). Neutrophils were identified by S100A9 expression; lack of vimentin coexpression distinguished neutrophils from stromal cells (*SI Appendix*, Fig. S1*G*). Tissue infiltration of neutrophils is a defining feature of an inflammatory reaction. Moreover, downregulation of *Casp8* led to an enlargement of the spleen, indicative of systemic inflammation (*SI Appendix*, Fig. S2*C*). This is consistent with previous reports that Caspase-8 loss leads to severe inflammatory skin disease (18) and chronic inflammation of the liver (23) and intestine (24).

To obtain more information about the different immune cell subpopulations affected by *Casp8* deletion, we performed flow cytometric analysis in which we characterized T cells and myeloid cells in skin and tongue. The number of αβT-cells (CD3^+^ GL3^−^) was significantly increased in the skin of *Casp8*^−/−^ mice (*SI Appendix*, Fig. S2 *D* and *E*). Moreover, there was an increase in the number of helper T cells (CD3^+^ CD4^+^) and regulatory T cells (Tregs; CD3^+^ CD4^+^ FoxP3^+^). In contrast, comparison of the tongue epithelium between control and *Casp8*^−/−^ mice showed no difference in the number of αβT-cells, helper T cells, and Tregs (Fig. 2 *E* and *F*), which further supports our immunostaining results. γδT-cells (CD3^+^ GL3^+^) were present in the skin of wild type and *Casp8^−/−^* mice (*SI Appendix*, Fig. S2*D*), but were not detectable in the tongue (Fig. 2*E*). There was an increase in the number of macrophages (CD11b^+^ F4/80^+^), neutrophils (CD11b^+^ Ly6G^+^), and monocytes (CD11b^+^ Ly6C^+^) in both skin and tongue (Fig. 2 *G* and *H* and *SI Appendix*, Fig. S2 *F* and *G*).

These data indicate that *Casp8* deletion triggers an inflammatory reaction that results in an increased immune infiltrate in the skin and the tongue. In the tongue, the immune response favors an innate rather than an adaptive immune reaction.

Transepithelial migration of neutrophils is a defining feature of an active inflammatory state. CD47, a cell surface glycoprotein involved in increasing neutrophil transepithelial migration (25, 26), was up-regulated in *Casp8*^−/−^ tongue epithelium (*SI Appendix*, Fig. S2*H*), which is consistent with the neutrophil infiltrate observed on *Casp8* deletion.

We have previously reported that *Krt76*^−/−^ mice, which have reduced expression of tight junctions in the epidermis (27) but not in the tongue (28), show increased susceptibility to oral cancer and systemic inflammation that correlates with an increase in Tregs (28) in the oral cavity. In contrast, *Casp8*^−/−^ mice did not show increased oral Tregs (Fig. 2*F*), suggesting that the immune cell infiltrate and the mechanisms responsible for the inflammatory phenotype in *Casp8*^−/−^ mice are different from those of *Krt76*^−/−^ mice.

### Activation of Autophagy Phenocopies Downregulation of Casp8.

**Fig. 3.**
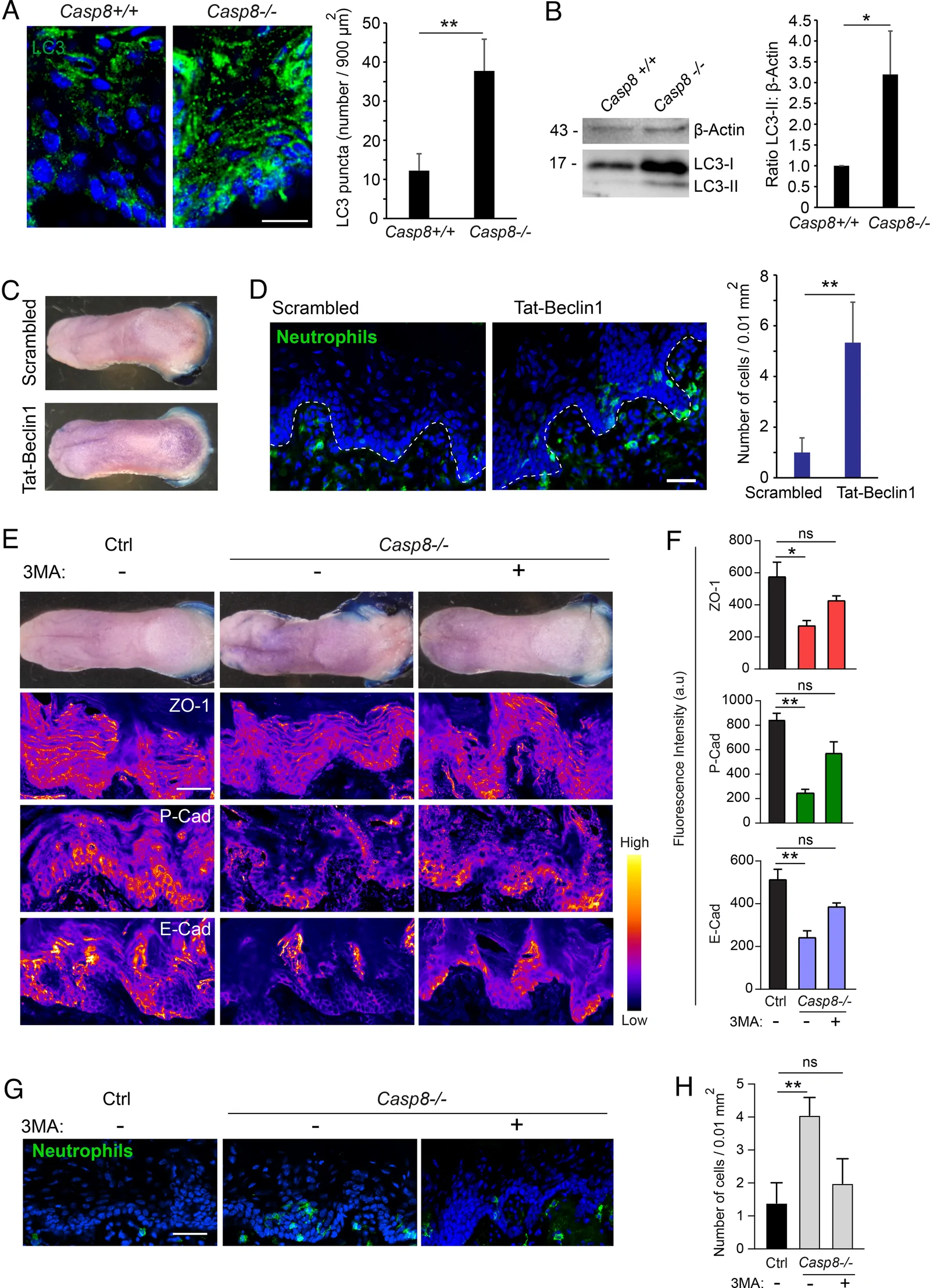
Activation of autophagy phenocopies *Casp8* deletion. (*A*) Representative tongue sections stained for LC3 (*Left*). Bar graphs showing the number of LC3+ puncta (*Right*). Data are the mean ± SD n = 3 mice per condition. (Scale bar, 20 µm.) (*B*) Tongue epithelium extracts from Ctrl and *Casp8*^−/−^ mice were analyzed by immunoblotting with antibodies to β-Actin and LC3 (*Left*). The position of molecular mass markers (kDa) is shown on the *Left*. The ratio LC3-II/ β-Actin was quantified (*Right*). Data are the mean ± SD from three independent experiments. (*C*) Tongues from Tat-Beclin 1 and scrambled control peptide-treated wild-type mice were stained with toluidine blue. (*D*) Tongue sections from Tat-Beclin 1 and scrambled control peptide-treated wild-type mice were stained with S100A9 antibody (*Left*). Bar graphs showing the number of neutrophils per 0.01 mm^2^ of tongue epithelium in Tat-Beclin1 and scrambled control peptide-treated mice (*Right*). Data are the mean ± SD n = 3 mice per condition. (Scale bar, 40 µm.) (*E*) Representative tongues (*Top*) and tongue sections of *Casp8*^+/+^ and *Casp8*^−/−^ mice treated (+) or not (−) with 3-MA were stained with ZO-1, P-Cad, and E-Cad antibodies and color-coded for signal intensity with ImageJ. (Scale bar, 40 µm.) (*F*) Bar graphs showing fluorescence intensity of ZO-1, P-Cad, and E-Cad. Data are the mean ± SD *n* = 3 mice per condition. (*G*) Representative tongue sections of *Casp8*^+/+^ and *Casp8*^−/−^ mice treated (+) or not (−) with 3-MA were stained with anti-S100A9. (Scale bar, 40 µm.) (*H*) Bar graphs showing the number of infiltrating neutrophils per 0.01 mm^2^ of tongue epithelium. Data are the mean ± SD n = 3 mice per condition. Two-tailed Student’s unpaired *t* test was used to determine statistical significance in *A*, *B*, and *D*. One-way ANOVA with Šidák’s multiple comparisons test was used to determine statistical significance in *F* and *H*. *P < 0.05; **P < 0.01; ns, not significant. The dashed lines demarcate epithelial-stroma boundaries.

Macroautophagy, more commonly referred to as autophagy, is a cellular recycling program that has been shown to regulate immune responses (29). The recycled cytoplasmic material is sequestered into a double-membrane vesicle, the autophagosome, which can be identified by microtubule-associated protein light chain 3B (LC3) puncta (30). In the last decade, Caspase-8 has been linked to autophagy. The oral epithelium of *Casp8*^−/−^ mice showed an increase in the number of LC3+ puncta that correlated with an increase in LC3 protein levels (Fig. 3 *A* and *B*), indicating that autophagy was induced on *Casp8* deletion. There were also increased LC3 puncta in the epidermis (*SI Appendix*, Fig. S3*A*).

To investigate whether autophagy was involved in epithelial integrity and the immune infiltrate, we used a cell-penetrant autophagy-inducing peptide, Tat-Beclin 1, which activates the autophagy regulator Beclin 1. Wild-type (WT) mice treated with Tat-Beclin 1 showed a defect in epithelial barrier integrity in the tongue that correlated with disruption in adherens junctions but not tight junctions (Fig. 3*C* and *SI Appendix*, Fig. S3 *B* and *C*). Tat-Beclin 1 treatment led to an increased neutrophil infiltrate in the tongue of WT mice (Fig. 3*D*). These results resembled the observations made in *Casp8*^−/−^ mice, and thus activation of autophagy phenocopies *Casp8* deletion.

To explore the potential link between autophagy and Caspase-8 in regulating epithelial integrity in the tongue, we treated *Casp8^−/−^* mice with the autophagy inhibitor 3-methyladenine (3-MA). Treatment with 3-MA partially restored the barrier defect caused by *Casp8* deletion (Fig. 3 *E* and *F* and *SI Appendix*, Fig. S3*D*), suggesting that Caspase-8 regulates epithelial integrity through autophagy. Furthermore, autophagy inhibition led to a reduction in the neutrophil infiltrate in the tongue of *Casp8*^−/−^ mice (Fig. 3 *G* and *H*). These data are consistent with a recent report showing that upon acute inflammation autophagy regulates neutrophil transendothelial migration (31).

### Casp8-Deficient Mice Show Increased Susceptibility to Oral Carcinogenesis.

**Fig. 4.**
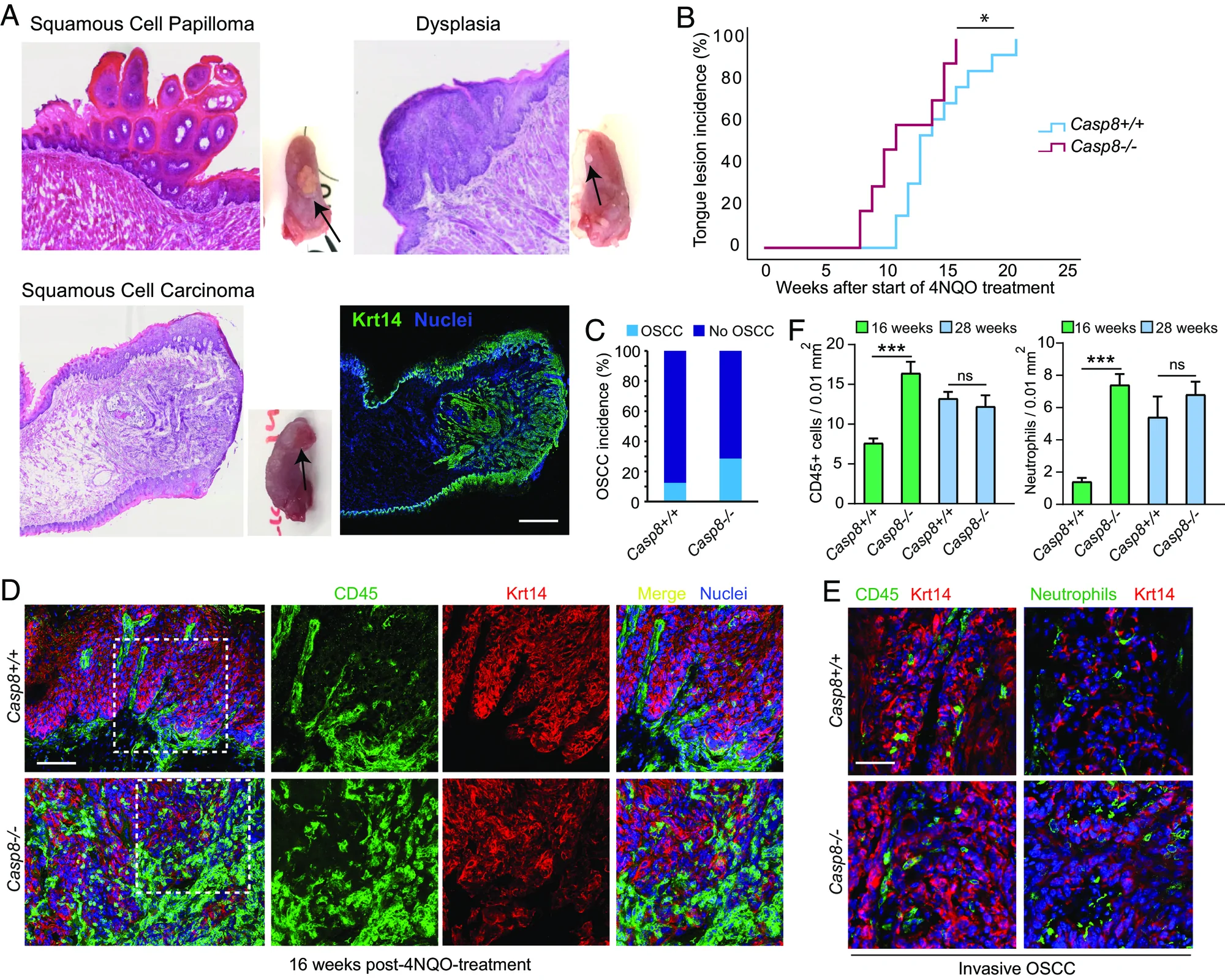
Caspase-8 regulates tongue tumor onset in 4NQO-treated mice. (*A*) Representative H&E staining and photographs of different types of oral lesion observed during 4NQO treatment (squamous cell papilloma, dysplasia, OSCC). OSCC stained with anti-Krt14 (*Bottom Right*). (Scale bar, 40 µm.) (*B*) Tongue lesion incidence in control (n = 13) and *Casp8*^−/−^ (n = 17) mice. (*C*) Bar graph showing the percentage of control (n = 1/8) and *Casp8*^−/−^ (n = 4/14) mice with OSCC 16 wk post-4NQO-treatment. (*D* and *E*) Representative OSCC sections stained for CD45 and Krt14 16 wk post-4NQO-treatment (*D*), and CD45, S100A9, and Krt14 28 wk post-4NQO-treatment (*E*). (*F*) Bar graphs showing the number of CD45+ cells (*Left*) and S100A9+ cells (*Right*) infiltrating the epithelium (*E*) at weeks 16 and 28 post-4NQO treatment. Data are the mean ± SD n = 3 mice per condition. (Scale bars, 40 µm.) The Mantel-Cox test was used to determine statistical significance in *B*. One-way ANOVA with Šidák’s multiple comparisons test was used to determine statistical significance in *F*. *P < 0.05; ***P < 0.001; ns, not significant.

To examine the effect of *Casp8* deletion on tumor formation we treated WT and *Casp8^−/−^* mice with the synthetic carcinogen 4-nitroquinoline-1-oxide (4NQO), which captures the heterogeneity and many of the hallmarks of human OSCC, mimicking the carcinogenic effects of tobacco and alcohol ingestion (32). Mice were given 4NQO in the drinking water for 16 wk and monitored for a total of 28 wk, as described previously (28). The lesions caused by 4NQO were assessed in the tongue by visual inspection and end-point histology (Fig. 4*A*). As reported previously, lesions progressed from hyperplasia to dysplasia to OSCC (Fig. 4*A*). In addition, we observed oral squamous cell papillomas, which had characteristic finger-like protrusions (Fig. 4*A*).

20% of the *Casp8*^−/−^ mice showed visible lesions 8 wk after the start of 4NQO treatment, whereas WT mice showed no lesions until week 11 posttreatment (Fig. 4*B*). 100% of the *Casp8^−/−^* and WT mice developed tongue lesions by 16 and 21 wk, respectively (Fig. 4*B*). The average onset of lesions was at 12 wk after treatment (n = 17) in *Casp8*^−/−^ mice, compared to 14 wk in control mice (n = 13). 21 wk posttreatment, with 100% of the mice showing at least one lesion, the percentage of mice with OSCCs was higher in *Casp8*^−/−^ mice (Fig. 4*C*). This suggests that loss of Caspase-8 is involved in tumor onset. There was no difference in tumor development between male and female mice. None of the mice developed spontaneous tumors in the absence of 4NQO treatment. Since *Casp8^−/−^* mice exhibit barrier defects, we speculate that in the carcinogen-induced tumor model, there is an increase in the penetration rate of 4NQO when compared with WT mice, which is consistent with our previous report in human OSCC lines that inactivating mutations in *CASP8* promote OSCC growth in culture by reducing intercellular adhesion (3).

We did not find any differences in the histology of individual WT and *Casp8*^−/−^ tumors (e.g., poorly or well differentiated) when comparing different tumor stages. Immune cells in *Casp8*^−/−^ mice had infiltrated the 4NQO-treated epithelium at week 16 posttreatment, whereas no substantial infiltration was found in control mice (Fig. 4 *D*–*F*). The increase in CD45+ cells in tumors of *Casp8*^−/−^ mice correlated with an increased number of neutrophils at week 16. However, when we compared immune infiltration in control mice and *Casp8*^−/−^ mice in invasive OSCCs, 28 wk posttreatment, no difference was observed (Fig. 4 *D*–*F*). The immune infiltrate included a large number of neutrophils, consistent with their role in promoting tumorigenesis (Fig. 4 *E* and *F*). This suggests that neutrophils play a role in tumor initiation rather than in tumor development.

We conclude that *Casp8* deletion reduces the time to tumor formation rather than the type of tumor that is formed.

### Delay in Tumorigenesis and Reduced DNA Damage Following Neutrophil Depletion.

**Fig. 5.**
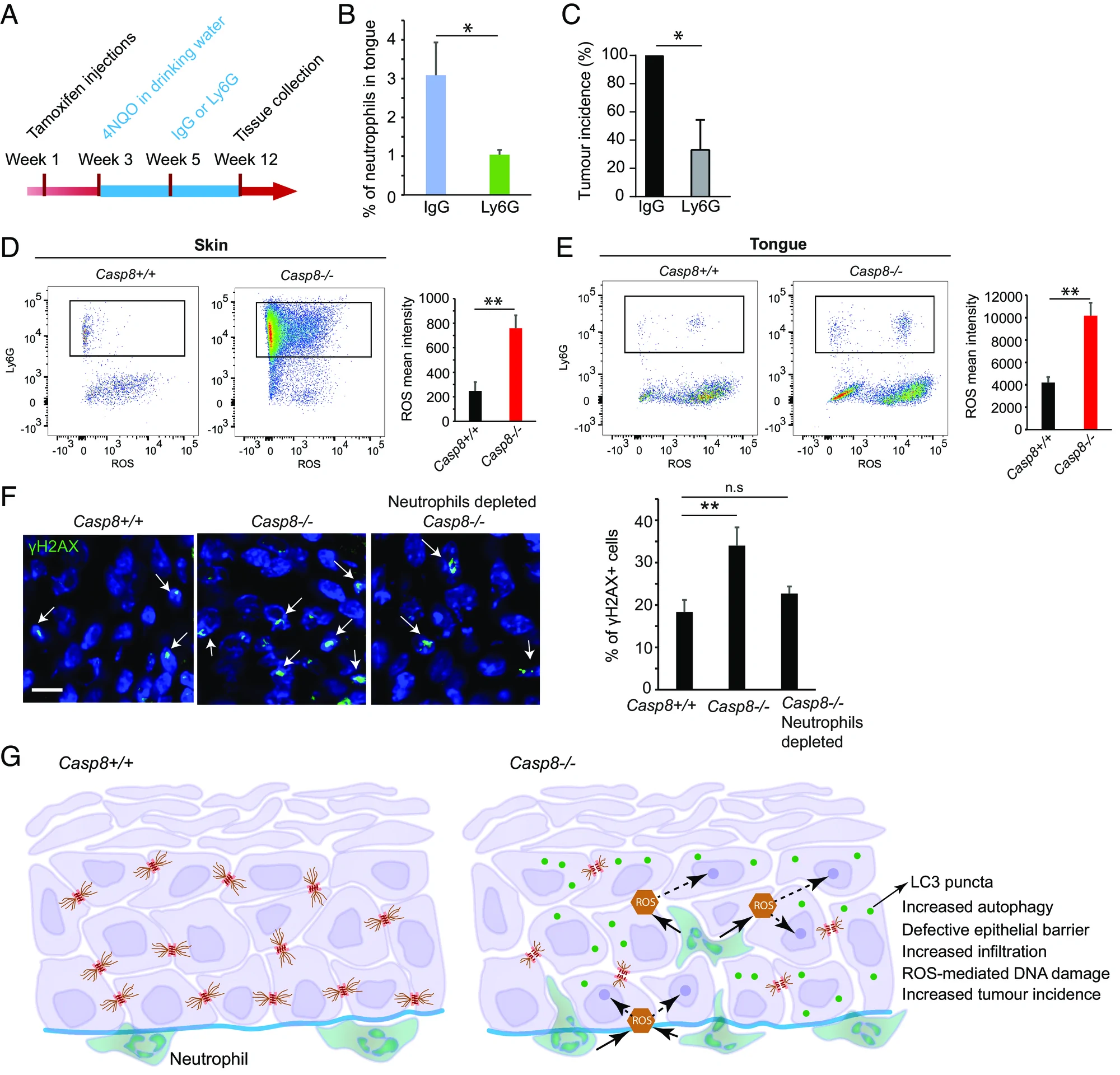
Neutrophils act as tumor promoters via ROS activity. (*A*) Schematic of the experimental design to test the impact of neutrophils in 4NQO-induced oral carcinogenesis. (*B*) Bar graph from flow cytometric analysis showing the percentage of neutrophils (CD11^+^ Ly6G^+^) in anti-Ly6G and IgG control treated mice. (*C*) Bar graph showing the tumor incidence in control (IgG, n = 6) and neutrophil depleted (Ly6G, n = 6) *Casp8^−/−^* mice. Data are the mean ± SD. (*D* and *E*) Representative flow cytometric analysis showing ROS activity in neutrophils of skin (*D*) and tongue (*E*) of control and *Casp8^−/−^* mice (*Left*). Bar graphs showing ROS mean intensity (*Right*). Data are the mean ± SD n = 3 mice per condition. (*F*) Representative OSCC sections of Ctrl, *Casp8*^−/−^, and *Casp8*^−/−^ neutrophil-depleted mice stained for γH2AX (*Left*). Bar graph showing the percentage of γH2AX+ cells in the indicated conditions (*Right*). Data are the mean ± SD n = 3 mice per condition. (Scale bar, 20 µm.) Two-tailed Student’s unpaired *t* test was used to determine statistical significance in *B*–*F*. *P < 0.05; **P < 0.01; ns, not significant. (*G*) Schematic of the effects of depleting *Casp8* on oral carcinogenesis. On *Casp8* deletion, there is an epithelial barrier defect that results in neutrophil infiltration and release of ROS in the epithelium, which in turn is responsible for the increased tumor incidence.

In recent years, neutrophils have emerged as important regulators of cancer. Neutrophils have been reported to act as tumor promoters or tumor suppressors, depending on the tissue context (33). To investigate the contribution of neutrophils to tumor development in our model, we depleted neutrophils by systemic administration of an anti-Ly6G (or an IgG isotype control) in *Casp8*^−/−^ mice for 8 wk, starting 2 wk after initiation of 4NQO treatment (Fig. 5 *A* and *B*). This protocol was chosen because approximately 80% of *Casp8*^−/−^ mice had developed oral lesions by week 12 after the start of the 4NQO treatment (Fig. 4*B*). Neutrophil depletion with Ly6G antibody cannot be maintained over extended periods. Therefore, the neutrophils observed in the tongue following Ly6G treatment may represent a population that reconstituted under our experimental conditions. Nevertheless, we observed a significant local reduction in the number of neutrophils that correlated with a reduced tumor incidence (Fig. 5*C*). This suggests that neutrophils cooperate with 4NQO to enhance tumorigenesis.

Neutrophil blockade did not restore the tongue barrier integrity of *Casp8*^−/−^ mice (*SI Appendix*, Fig. S4*A*). Furthermore, neutrophil depletion had no effect on activation of autophagy induced by *Casp8* deletion (*SI Appendix*, Fig. S4*B*), suggesting that neutrophil recruitment is independent of autophagy induction.

Reactive oxygen species (ROS) activity in neutrophils is linked to cancer progression (16). We therefore examined ROS in neutrophils isolated from skin and tongue of control and *Casp8*^−/−^ mice and found that ROS activity was significantly greater in *Casp8* deficient mice than in control mice (Fig. 5 *D* and *E*). In the control group ROS levels were higher in the tongue than in skin, which may reflect the constant immune activation taking place in the oral cavity.

To investigate whether ROS activity correlated with DNA damage in epithelial cells, we stained WT and *Casp8*^−/−^ OSCCs with an antibody to histone H2AX phosphorylation (γH2AX) (34). OSCCs of *Casp8*^−/−^ mice showed higher levels of DNA damage compared to WT mice (Fig. 5*F*). Remarkably, the increase in DNA double-strand breaks (γH2AX foci) enhanced by *Casp8* knockout was abolished in neutrophil-depleted OSCCs (Fig. 5*F*), indicating that neutrophils in the OSCC microenvironment affect the susceptibility of the tissue to 4NQO. This is in line with a recent study showing that neutrophils amplify carcinogen-induced DNA damage in the lung (35).

## Discussion

During tumor development, tissue-specific alterations in the microenvironment contribute to tumor behavior. In the present study, we have examined the effects of depleting *Casp8* in the oral epithelium. The tongue epithelium of *Casp8*^−/−^ mice showed no histological or proliferative changes. *Casp8*^−/−^ mice, however, developed a defective oral epithelial barrier and an inflammatory infiltrate with a high proportion of neutrophils. Mechanistically, Caspase-8-dependent autophagy was linked to epithelial integrity and neutrophil infiltration, the latter enhancing the tissue susceptibility to developing tumors (Fig. 5*G*).

The relationship between chronic inflammation and cancer is well established; however, the mechanisms are still unresolved (36). The *Casp8*^−/−^ mouse serves as a model to study this link, which complements our previously reported *Krt76*^−/−^ mouse model. In contrast to the *Krt76*^−/−^ model, in which increased tumor incidence was not due to epithelial barrier malfunction but to an accumulation of Tregs in the oral epithelium (28), the *Casp8*^−/−^ mouse model showed defective epithelial barrier formation and an immune infiltrate enriched in neutrophils. Moreover, we previously reported another mouse model of inflammation-mediated skin tumorigenesis in which constitutively active MEK1 is expressed in the suprabasal layers of the epidermis. In that model, macrophage infiltration promotes skin carcinogenesis (37). The MEK mouse model phenocopies the effect of epidermal *Casp8* deletion, stimulating proliferation of Krt14+ basal keratinocytes through IL-1α (38, 39). In contrast, another mouse model deficient in envoplakin, periplakin, and involucrin (Epi^−/−^ mice), three components of the epidermal barrier on which the cornified envelope assembles, have a defective epidermal barrier that is protective against skin cancer (40). In the Epi^−/−^ model there is an increase in neutrophil infiltration that does not correlate with epidermal hyperplasia (40). Together, these data suggest that inflammation can either promote or suppress epithelial tumorigenesis depending on the immune cells involved.

Failure to resolve chronic inflammation is one of the initial triggers of carcinogenesis (41), and neutrophils have been shown to induce genomic instability, impeding resolution of inflammation and wound healing (42). Our results suggest that the local microenvironment of the *Casp8*-depleted OSCCs resembles a state of chronic inflammation, with barrier malfunction and accumulation of tumor-promoting neutrophils. This is consistent with previous reports that the loss of epidermal Caspase-8 simulates a wound-healing response (19). These observations, together with our finding that neutrophils are involved in DNA damage during carcinogenesis, lead us to speculate that neutrophils in the tongue of *Casp8^−^*^/−^ mice drive a tissue-dependent inflammatory microenvironment that persists during tumor development. Neutrophils are not the only immune cell type that infiltrates the epithelium, and thus further research will be required to understand the extent to which neutrophils influence this inflammatory reaction compared to other immune cell populations.

In *Casp8*-depleted mice, leukocytes infiltrate several organs, including the lung, pancreas, and pleura (18), and thus some effects observed in the tongue are likely to be attributable to systemic inflammation rather than to a local effect. Another intriguing possibility is that the neutrophils that have been involved in tissue repair caused by *Casp8* deletion undergo reverse transmigration from the site of injury and reenter the vasculature (43, 44) ending up in the tongue where no injury was observed (Fig. 1*B*). Moreover, neutrophils are highly heterogeneous, with distinct functional capabilities (16, 45), although it has been recently reported that once they have entered the tumor, they converge into a single cell subset (45). This raises the question of whether reprogramming to a single cell state is conserved or tissue-dependent. Whether the neutrophils in our *Casp8*^−/−^ mouse model have the ability to promote tumorigenesis themselves will require further investigation.

4NQO induces an immunosuppressive response during OSCC development that mainly affects B-lymphocytes and γδ T-cells (46), and neutrophils have been shown to have an immunosuppressive function that hinders antitumor immune responses (16). Furthermore, we recently reported that human OSCCs with low activity of Caspase-8 have apoptotic tumor infiltrating lymphocytes (47), in line with reports that *CASP8* mutations lead to human immunodeficiency (48). Therefore, it is plausible that 4NQO and neutrophils cooperate in tumor progression by inducing immunosuppressive mechanisms. This would explain why 4NQO and neutrophils have cumulative effects on tumorigenesis.

The role of autophagy in regulating inflammation is well established. Moreover, several studies have reported a link between autophagy and Caspase-8 (11, 12, 49). We show that activation of autophagy phenocopies *Casp8* deletion (Fig. 3). The induction of autophagy correlated with epithelial barrier defects and neutrophil recruitment, suggesting a role for autophagy in modulating epithelial integrity. This is consistent with a recent report showing that autophagy regulates cell adhesion molecules and remodeling of endothelial cell junctions (31). Further investigation will be required to determine the extent of cooperation between autophagy and neutrophils in initiating tumorigenesis.

Although defects in the epidermal barrier, mutations in *Casp8* and individual components of the immune system have previously been associated with cancer, our model links defective epidermal barrier to autophagy activation and cancer susceptibility. Our study provides a framework for further analysis of the association between immune infiltration and cancer incidence and highlights the importance of therapeutic strategies that target specific tumor microenvironment components.

## Materials and Methods

### Animal Procedures.

All mouse procedures were subjected to local ethical approval at King’s College London (UK) and performed under a UK Government Home Office license (PP70/8474 or PP0313918). Casp8^fl/fl^ mice (19) were obtained from The Jackson Laboratory (#027002). Mice in which Casp8 was deleted via the Krt14 promoter were generated by crossbreeding Casp8^fl/fl^ mice with Krt14CreER mice (50). All mice used in the experiments were on a C57BL/6J background. Experiments were carried out with male and female mice. No gender-specific differences in the phenotypes analyzed were observed. Deletion of *Casp8* in cells expressing *Krt14* was induced by intraperitoneal injection of Tamoxifen into 8 to 10-wk-old mice (*Casp8^−/−^*). Mice received a dose of 20 mg/mL Tamoxifen in corn oil for four consecutive days. Littermates negative for *Cre* were used as controls. To induce autophagy, 6-wk-old mice were injected intraperitoneally with Tat-scrambled peptide (Bio-Techne) or Tat-beclin 1 (Biotechne, 20 mg kg^−1^) in phosphate buffered saline (PBS). Mice received a daily dose for 2 wk. Tissue was collected at the indicated time points and embedded in optimal cutting temperature (OCT) compound prior to immunostaining. 3-MA (Sigma-Aldrich) was used to inhibit autophagy. Two weeks after *Casp8* deletion, *Casp8^−/−^* mice were injected intraperitoneally daily with 15 mg/kg of 3-MA for 3 wk. Control mice were treated with PBS.

### Immunofluorescence Staining and Histology.

Mouse tissue samples were processed and stained essentially as described previously (51). OCT-embedded frozen sections were fixed in 4% paraformaldehyde/PBS pH 7.4, permeabilized with 0.1% Triton X-100/PBS for 10 min at room temperature and blocked for 1 h at room temperature in 10% fetal bovine serum (Sigma-Aldrich), 3% bovine serum albumin (Sigma-Aldrich), 0.02% fish skin gelatin (Sigma-Aldrich), 0.05% Triton X-100, and 0.05% Tween 20 (Sigma-Aldrich) in PBS. Sections were incubated overnight at 4 °C with primary antibodies: CD45 (30-F11 clone, eBioscience), CD3 (BioLegend), CD68, Krt14 (Covance), S100A9 (2B10 clone, Abcam), yH2AX (Sigma-Aldrich), Ki67 (Novus Biologicals), CD47 (Bio X Cell), LC3 (MBL International), Caspase-8 (Cell Signaling), ZO-1 (Invitrogen), P-Cad (R&D Systems), E-Cad (24E10, Cell Signaling), Filaggrin (Abcam), or Vimentin (Abcam). Following washing, sections were labeled for 1 h at room temperature with secondary antibodies (Invitrogen): Donkey anti-Rabbit IgG Alexa Fluor 647, Donkey anti-Rat IgG Alexa Fluor 647, Goat anti-Chicken Alexa Fluor 488, or Goat anti-Rabbit IgG Alexa Fluor 488. Alexa 488 phalloidin (1:500, Invitrogen) was used to label F-actin. DAPI (Thermo Fisher Scientific) was used to counterstain nuclei. Sections were mounted with ProLong Gold Antifade Mountant (Thermo Fisher). Images were obtained using a Nikon A1R confocal microscope (Nikon) with 20× or 40× objectives and analyzed with ImageJ (NIH). For hematoxylin and eosin staining, tissues were embedded in OCT, sectioned, and postfixed in 4% paraformaldehyde/PBS pH 7.4 for 10 min before staining by conventional methods. For each mouse, 3 to 5 sections were analyzed, and measurements from the analyzed fields were averaged to obtain a single value for each mouse. For Fig. 3*H*, a subsequent blinded reanalysis was performed using anonymized images, with the investigator blinded to the experimental conditions during quantification. Images were acquired using a NanoZoomer 2.0RS Digital Slide Scanner (Hamamatsu Photonics K.K.) and analyzed using NPD viewer software (Hamamatsu).

### Flow Cytometry.

Single cells were labeled as described in ref. 51. To isolate skin cells, the skin was minced finely and resuspended in 3 mL of digestion mix (2 mg/mL collagenase XI, 0.5 mg/mL hyaluronidase, and 0.1 mg/mL DNase in Roswell Park Memorial Institute (RPMI) medium with 1% (4-(2-hydroxyethyl)-1-piperazineethanesulfonic acid (HEPES), 1% penicillin-streptomycin, and 10% fetal bovine serum). The skin was then incubated in a shaking incubator at 37 °C at 250 rpm for 45 min. Following resuspension in 20 mL of RPMI/HEPES/P-S/FBS medium the digested skin was passed through 100 µm and 40 µm cell strainers. The resulting cell suspension was then pelleted and resuspended in PBS for antibody labeling.

To isolate tongue cells, the tongue was minced in digestion medium containing collagenase type II (2 mg/mL, Worthington Biochemicals) and DNase I (1 mg/mL, Roche) solution in PBS with 2% (vol/vol) fetal bovine serum. The tissue was then digested for 25 min at 37 °C in a shaker bath at 255 rpm. 20 µL of 0.5 mM ethylenediaminetetraacetic acid per 2 mL sample was added to the digested tissue and incubated for an additional 10 min. Cells were washed and filtered through a 70-µm strainer and then resuspended in PBS for labeling with the indicated antibodies.

Strict dead cell and doublet cell exclusion criteria were included for all immune cell analysis, followed by pregating for all hematopoietic cells as CD45+. The following antibodies were used: CD3-BV711 (BioLegend), CD8-BV605 (BioLegend), CD4-BV650 (BioLegend), FoxP3-violet 450/50, CD45-AlexaFluor700 (eBioscience), CD11b-APC-Cy7 (BioLegend), Ly6G-BUV395 (BD Biosciences), Ly6C-FITC (BD Biosciences). All samples were run on a Fortessa 2 (BD Biosciences) at King’s College London Biomedical Research Centre Flow Cytometry Core. For compensation, UltraComp eBeads were stained for each antibody following the same procedure as for cell staining. Data were analyzed using FlowJo software.

### Epithelial Permeability Assays.

As previously described (28), intact tongues and pieces of skin were dissected and dehydrated through a methanol series (25, 50, 75, and 100% methanol, 1 min per step), rehydrated in PBS, and then stained with 0.1% toluidine blue in water for 10 min at room temperature. Samples were washed in PBS and immediately imaged using a Nikon SWZ18 and Nikon DS-Ri2 camera.

### Neutrophil Depletion.

8 to 12-wk-old mice were treated with either an anti-Ly6G antibody (BioXCell, clone 1A8) or a rat IgG2a isotype control (BioXCell). Injections of 100 µL of antibody solution (12.5 µg/mouse in PBS) were administered intraperitoneally every second day for 8 wk during 4NQO treatment.

### 4NQO Carcinogenesis.

4NQO (Sigma, 100 µg/mL) was administered in the drinking water as the sole source of drinking water during the carcinogen-treatment period, as previously described (28). 4NQO-containing water was changed once a week for 16 wk. Once a week, 4NQO-treated mice were sedated with inhaled isoflurane for oral lesion examination.

### ROS Determination.

ROS activity was measured using the total ROS assay kit 520 nm (Thermo Fisher Scientific). Skin and tongue cell suspensions were incubated with ROS assay stain for 1 h at 37 °C, stained with an anti-Ly6G antibody, and analyzed by flow cytometry.

### Tongue Epithelium Extraction.

Tongue epithelium was isolated by injecting tongues from killed mice with an enzymatic cocktail consisting of collagenase A (1 mg/mL) and Dispase II (2 mg/mL). Tongues were subsequently incubated at 37 °C for 30 min. The epithelium was separated from the underlying connective tissue using tweezers.

### Real-Time qRT-PCR.

Total RNA extraction from tongue epithelium and epidermis and complementary DNA synthesis were performed using the Qiagen RNeasy Mini Kit (Qiagen) and the QuantiTect Reverse Transcription kit (Qiagen) respectively, according to the manufacturer’s instructions. Quantitative real-time reverse transcriptase polymerase chain reactions (qRT–PCR) were performed on a CFX384 Real-Time System (Bio-Rad Laboratories) using TaqMan Fast Universal PCR Master Mix (Life Technologies). Values were normalized to housekeeping genes (*Rn18*, *Gapdh,* and/or *Tbp*) and relative quantification of gene expression was performed using either the 2^−ΔCt^ or 2^−ΔΔCt^ method, as described previously (51). For each biological replicate, the reaction was performed in technical duplicates.

### Immunoblotting.

Samples from tongue epithelium were prepared in 4× Laemmli buffer and denatured at 95 °C for 5 min. Gel electrophoresis was performed in 10 to 12% SDS-PAGE gels. Proteins were transferred onto polyvinylidene fluoride membranes using the Turbo Transfer system at 13 V for 7 min. Blocking was performed in PBS containing 5% nonfat milk and 0.1% Tween 20 at room temperature for 1 h. Primary antibodies were incubated overnight at 4 °C. After extensive washes, the membranes were incubated with horseradish peroxidase-conjugated secondary antibodies at room temperature for 1 h. Membranes were then washed before ECL development. ECL signal was acquired using a Chemidoc (Bio-rad).

### Statistical Analysis.

Statistical analysis was performed with GraphPad Prism software. Data are expressed as the mean ± SD of at least three independent experiments. Statistical significance was determined with the two-tailed Student’s unpaired *t* test, ordinary one-way ANOVA with Šidák’s multiple comparisons test or Mantel-Cox test. No statistical methods were used to predetermine sample sizes. Data collection and analysis were not performed blind to the conditions of the experiments.

## Supplementary Material

Appendix 01 (PDF)

## Data Availability

All study data are included in the article and/or *SI Appendix*.
