# Supplementary material for: Caspase-8-dependent autophagy regulates neutrophil infiltration in oral squamous cell carcinoma
Source: Proc Natl Acad Sci U S A. 2024 Dec 3;121(50):e2406944121. doi: 10.1073/pnas.2406944121 (PMC11648635; doi:10.1073/pnas.2406944121)
Supplement: Supplementary file 1 — Appendix 01 (PDF) [file pnas.2406944121.sapp.pdf]

Supporting Information for

Caspase-8-dependent autophagy regulates neutrophil infiltration in oral squamous cell carcinoma

Miguel Bernabé-Rubio[1] and Fiona M. Watt[1,2*]

*Corresponding author: fiona.watt@embo.org (F. M. Watt)

This PDF file includes:

Figures S1 to S4

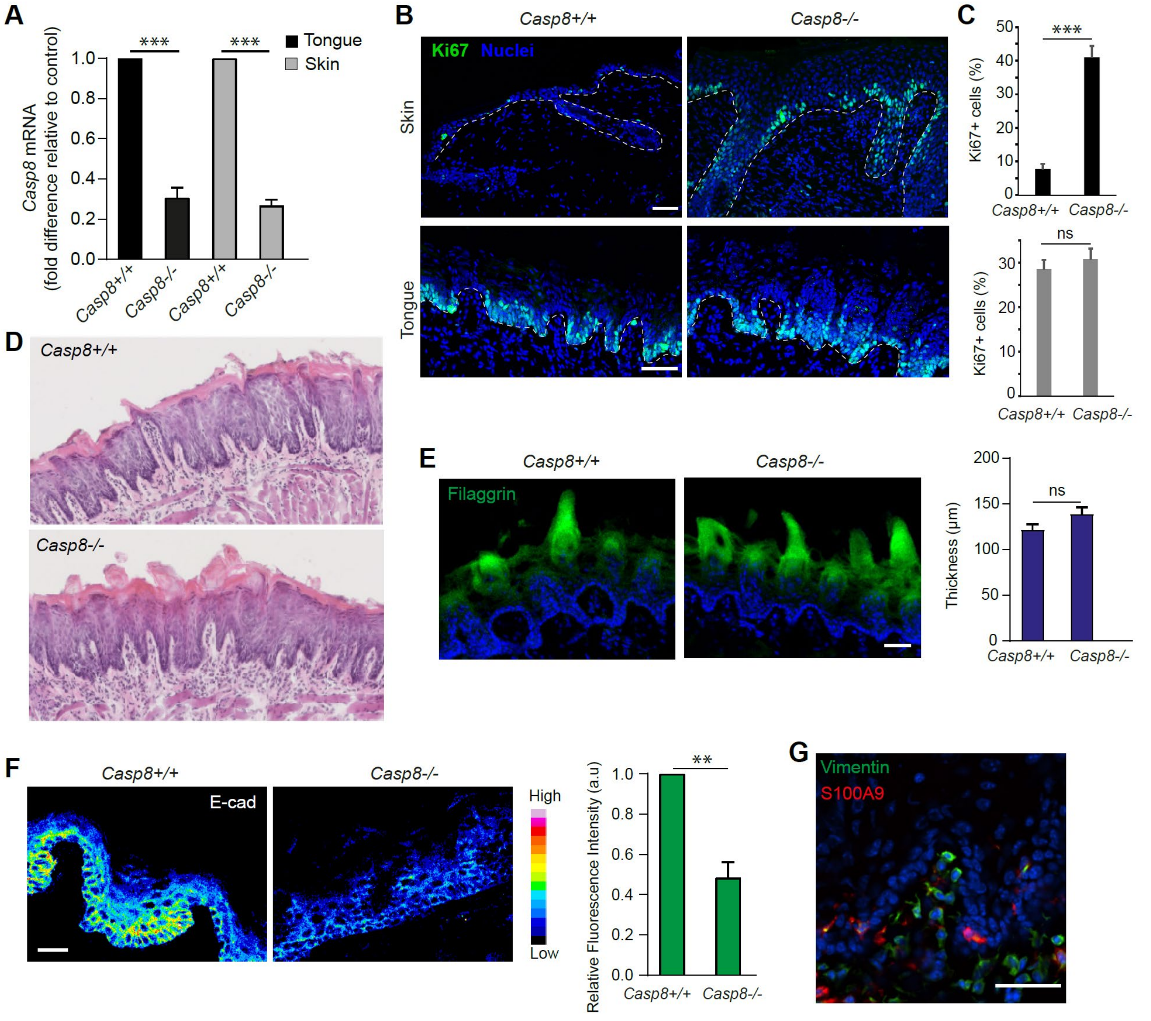

Fig. S1. *Casp8* deletion does not affect the tongue architecture. (A) Bar graphs showing expression of *Casp8* measured by RT-qPCR. Data are the mean ± s.d. from three independent experiments. **(B)** Representative skin (top) and tongue (bottom) sections stained for Ki67. Scale bars, 40 µm. **(C)** Bar graphs showing the percentage of Ki67+ cells in skin (top) and tongue (bottom). Note that the number of proliferating cells in the tongue remains unchanged on *Casp8* deletion. Data are the mean ± s.d. n=3 mice per condition. **(D)** Representative H&E staining of the tongue of Ctrl and *Casp8*$^{-/-}$ mice. **(E)** Representative tongue sections stained with antibodies to Filaggrin (left). Thickness of the Filaggrin layer (right). Data are the mean ± s.d. from three independent experiments. **(F)** Representative tongue sections stained for E-Cad and color-coded for signal intensity with ImageJ (left). Bar graph showing the relative fluorescence intensity (a.u) in Ctrl and *Casp8*$^{-/-}$ mice (right). Data are the mean ± s.d. from three independent experiments. **(G)** Representative tongue section stained with Vimentin and S100A9 antibodies. Two-tailed Student's unpaired *t*-test was used to determine statistical significance in (A), (C), (E) and (F). $^{**}P<0.01$; $^{***}P<0.001$; ns, not significant.

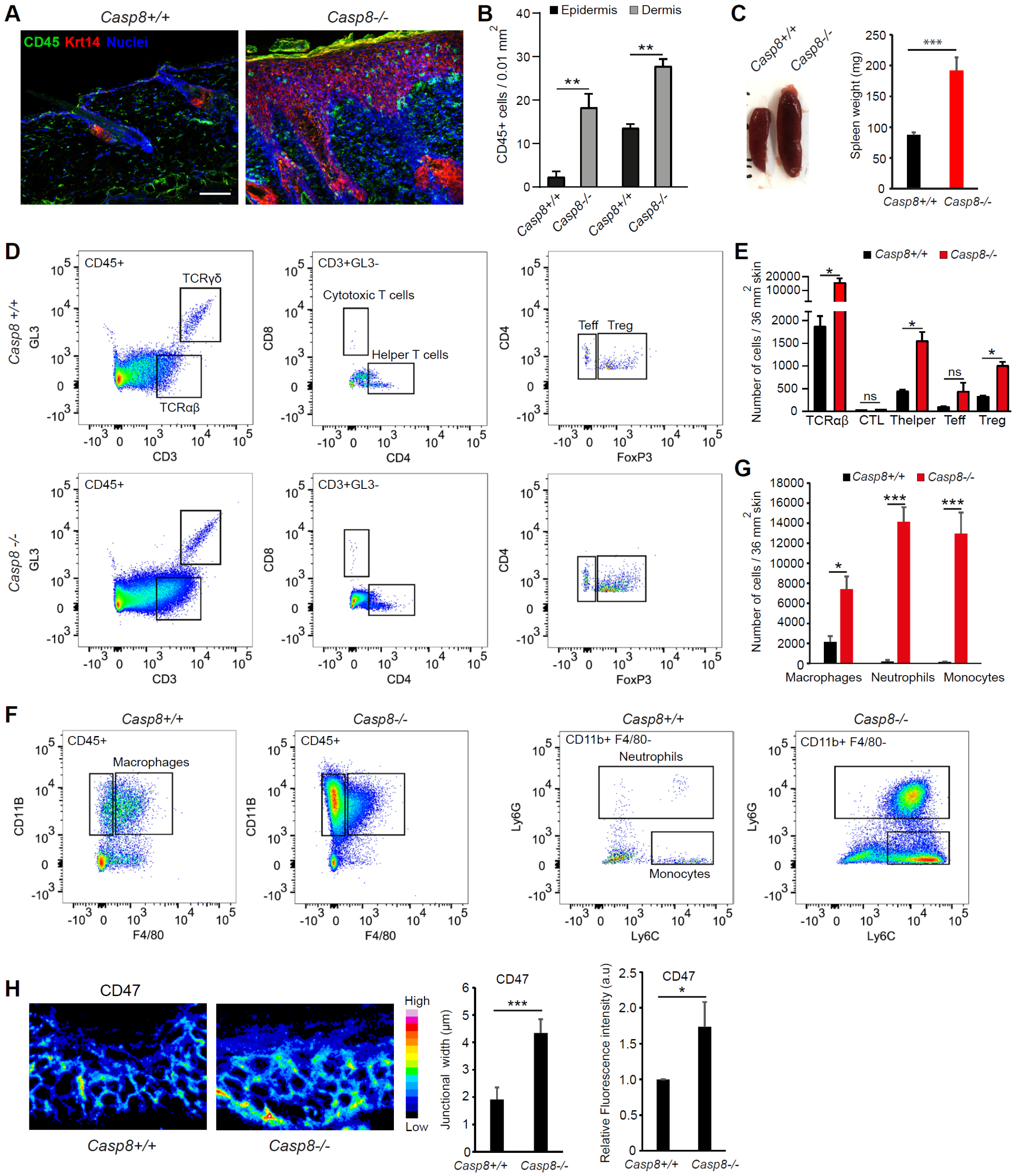

Fig. S2. Inflammatory response to Casp8 deletion. (A) Representative skin sections stained for CD45. Scale bar, 40 µm. **(B)** Bar graphs showing the number of CD45+ cells per 0.01 mm^2 in epidermis and dermis. Data are the mean ± s.d. n=5 mice per condition. **(C)** Representative image of spleens of *Casp8*$^{+/+}$ and *Casp8*$^{-/-}$ mice (left). Bar graphs showing the spleen weight (mg) of Ctrl and *Casp8*$^{-/-}$ mice (right). Data are the mean ± s.d. n=5 mice per condition. **(D)** Representative flow cytometric analysis of αβT-cells ($CD3^+$ $GL3^-$), γδT-cells ($CD3^+$ $GL3^+$), cytotoxic T cells ($CD3^+$ $CD8^+$), helper T cells ($CD3^+$ $CD4^+$), effector T cells ($CD3^+$ $CD4^+$ $FoxP3^-$) and Tregs ($CD3^+$ $CD4^+$ $FoxP3^+$) in skin of *Casp8*$^{-/-}$ and control mice. **(E)** Bar graphs showing the number of cells analyzed in (D).

Data are the mean ± s.d. n=2 mice per condition. **(F)** Representative flow cytometric analysis of macrophages ($CD11b^+$ $F4/80^+$), neutrophils ($CD11b^+$ $Ly6G^+$) and monocytes ($CD11b^+$ $Ly6C^+$) in skin of *Casp8*$^{-/-}$ and Ctrl mice. **(G)** Bar graphs showing the number of cells analyzed in (F). Data are the mean ± s.d. n=3 mice per condition. **(H)** Representative tongue section stained for CD47 and color-coded for signal intensity with ImageJ (left). Bar graphs showing the junctional width (µm, middle) and relative fluorescence intensity (a.u.) (right). Data are the mean ± s.d. n=3 mice per condition. Two-tailed Student's unpaired *t*-test was used to determine statistical significance in (B), (C), (E), (G) and (H). *P<0.05; **P<0.01; ***P<0.001; ns, not significant.

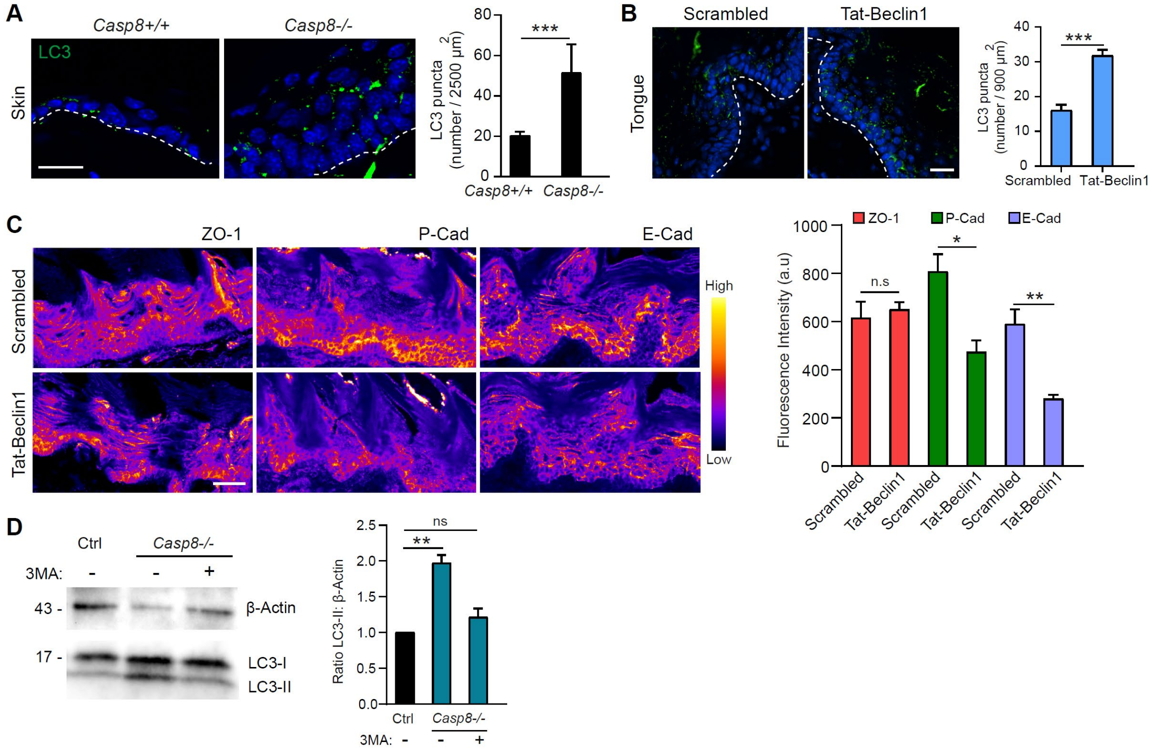

Fig. S3. Effects of altering autophagy. (A) Representative skin sections (*Casp8*$^{+/+}$ and *Casp8*$^{-/-}$) stained for LC3 (left). Bar graph showing the number of LC3+ puncta. Data are the mean ± s.d. n=3 mice per condition. Scale bar, 20 µm. **(B)** Representative tongue sections from Tat-Beclin 1 and scrambled control peptide-treated wild-type mice stained for LC3 (left). Bar graph showing the number of LC3+ puncta. Data are the mean ± s.d. n=3 mice per condition. Scale bar, 20 µm. **(C)** Representative tongue sections from Tat-Beclin 1 and scrambled control peptide-treated wild-type mice were stained with ZO-1, P-Cad and E-Cad antibodies and color-coded for signal intensity with ImageJ. Scale bar, 40 µm. Bar graphs showing fluorescence intensity of ZO-1, P-Cad, and E-Cad per 0.01 mm^2 epithelium. Data are the mean ± s.d. $n = 3$ mice per condition. **(D)** Tongue epithelium extracts from Ctrl and *Casp8*$^{-/-}$ mice treated (+) or not (-) with 3-MA were analyzed by immunoblotting with antibodies to β-Actin and LC3 (left). The position of molecular mass markers (kDa) is shown on the left. The ratio LC3-II/ β-Actin was quantified (right). Data are the mean ± s.d. from three independent experiments. Two-tailed Student's unpaired *t*-test was used to determine statistical significance in (A) and (B). One-way ANOVA with Šidák's multiple comparisons test was used to determine statistical significance in (C) and (D). *P<0.05; **P<0.01; ***P<0.001; ns, not significant.

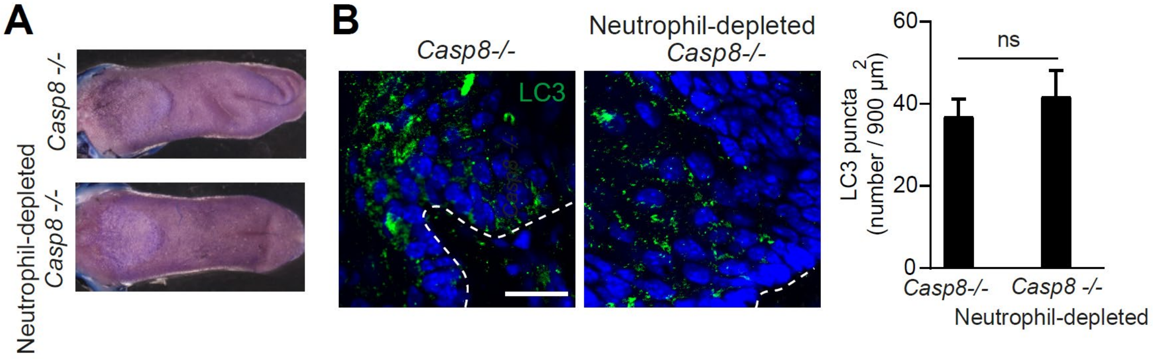

Fig. S4. Effects on tongue epithelium of depleting neutrophils

(A) Tongues from *Casp8*$^{-/-}$ mice treated with Ly6G antibody or an IgG2a isotype control were stained with toluidine blue. **(B)** Representative tongue sections of *Casp8*$^{-/-}$ mice treated with Ly6G antibody or an IgG2a isotype control were stained for LC3 (left). Bar graph showing the number of LC3+ puncta (right). Data are the mean ± s.d. n=3 mice per condition. Scale bar, 20 µm. Two-tailed Student's unpaired *t*-test was used to determine statistical significance in (B). ns, not significant.
